# The Effect of Vitamin D Level on Parathyroid Hormone and Alkaline Phosphatase

**DOI:** 10.3390/diagnostics12112828

**Published:** 2022-11-17

**Authors:** Hussein Abdullah Rajab

**Affiliations:** Najran University Hospital, Medical College, Najran University, Najran 66255, Saudi Arabia; harajab@nu.edu.sa; Tel.: +966-558-683-966

**Keywords:** 25-hydroxyvitamin D (25OHD), parathyroid hormone (PTH), alkaline phosphatase (ALP)

## Abstract

Over the years, we have noticed in our clinical practice that patients with 25-hydroxyvitamin D (25OHD) levels below 15 ng/mL are more symptomatic than those with higher levels. The aim of this research is to investigate changes in both parathyroid hormone (PTH) and alkaline phosphatase (ALP) at different vitamin D levels to determine if lower vitamin D levels are associated with more severe changes in PTH and ALP, which may explain the presence and severity of symptoms at those lower 25OHD levels. We looked for correlations between 25OHD level, PTH, and ALP in 1311 samples between 2015 and 2019 at our endocrine clinic to determine if vitamin D level correlates with changes in PTH and ALP. We further categorized vitamin D deficiency levels into three categories based on the severity of the reported symptoms. As expected, there were inverse but significant correlations between 25OHD, PTH, and ALP. The lower the 25OHD, the higher the PTH and ALP levels. When 25OHD was below 10 ng/mL, PTH was increased in 65% of the samples and ALP was elevated in 21% of the samples; however, PTH and ALP were normal in 70% and 87%, respectively, of patients with 25OHD levels between 15 < 20 ng/mL. The results support our clinical observations since most of the patients with 25OHD greater than 15 ng/mL had normal PTH and ALP, which may explain the lack of symptoms in these patients.

## 1. Introduction

Vitamin D deficiency is widespread worldwide. Clinically, vitamin D status is assessed by measuring levels of 25-hydroxyvitamin D (25OHD), a lipid-soluble vitamin with a long half-life. Total 25OHD is the sum of plant-based vitamin D2 (ergocalciferol) and animal-based vitamin D3, or cholecalciferol [1,2]. In 2011, the Institute of Medicine released its report regarding vitamin D recommendations based on bone-related health. The dietary reference intake (DRI) suggests that an estimated average intake (EAI) of 400 IU/d of vitamin D was assumed to achieve a vitamin D level of around 16 ng/mL in 50% of the population. The recommended daily allowance (RDA) for vitamin D is 600 IU/d for ages 1–70 and 800 IU/d for those over 70 years old. This is assumed to achieve vitamin D levels of around 20 ng/mL in 97% of the population [3]. The definition of vitamin D deficiency based on a blood level of 25OHD remains controversial within different medical societies. In an eight-week study, participants received 50,000 IU of vitamin D weekly, and calcium supplementation demonstrated a significant reduction in their PTH levels in those with an initial level of 25OHD below 25 ng/mL [3]. As shown in several—but not all—studies, there is an inverse relationship between 25OHD and PTH; when 25OHD reaches 30–40 ng/mL, PTH starts to plateau [3,4,5,6]. Thus, based on these and other studies, the Endocrine Society guidelines define vitamin D insufficiency as 25OHD of 20 to 30 ng/mL and deficiency as 25OHD below 20 ng/mL, whereas the American Association of Clinical Endocrinologists considers vitamin D deficiency as a 25OHD below 30 ng/mL and the optimal level between 30 and 50 ng/mL. The Institute of Medicine defines vitamin D deficiency as a 25OHD below 12 ng/mL, insufficiency as a 25OHD between 12 and 20 ng/mL, and the optimal level as a 25OHD above 20 mg/mL [2,3,5,6,7,8,9,10]. According to these definitions, around 20–100% of European and Northern American adults living in the community are vitamin-D-deficient [2,3,11,12,13,14].

In 2005–2006, an analysis of the National Health and Nutrition Examination Survey (NHANES) found that the overall prevalence rate of vitamin D deficiency was 41.6% among U.S. adults when using a cutoff of ≤20 ng/mL [7,8,9,10,11,12,13,14,15]. Black and Hispanic populations have significantly higher rates of vitamin D deficiency—(82.1%) and (69.2%), respectively—compared to white people. 

The vitamin D insufficiency threshold was based on the RDA of vitamin D to achieve 25OHD around 20 ng/mL in 97% of the population, and it was not based on biochemical or structural changes [3].

A recent study showed biochemical changes when 25OHD was below 10 ng/mL. However, there was no biochemical evidence when the levels were between 20–30 mg/mL to justify labeling patients with vitamin D insufficiency or to justify vitamin D supplementation in this category [16]. Although vitamin D deficiency is quite common in our area based on routine laboratory testing, we also have noticed that a majority of patients do not have symptoms, which made us consider whether the normal level in this area differs from that in the U.S. and Europe or whether the term “insufficiency” is clinically meaningful since it is not supported by clinical symptoms or signs or by biochemical or bone changes [16].

Based on years of clinical experience at our endocrine clinic, the 25OHD level does not predict symptoms according to these thresholds. Most of the patients have symptoms only when vitamin D is below 15 ng/mL. A further decline in vitamin D levels worsens the symptoms. These symptoms include fatigue, tiredness, muscle aches, proximal muscle weakness, difficulty standing and walking, bone pain, low back pain, cold intolerance, numbness and tingling, muscle spasms, hair loss, depression, mental fog, decreased concentration, and decreased memory [17,18]. It has been noticed that our patients’ symptoms improved significantly after the vitamin D course during their follow-up visits. Very low levels of vitamin D predict the severity of these symptoms. A study evaluated the correlation between vitamin D deficiency symptoms and the effect of vitamin D treatment on symptom severity. Symptoms include musculoskeletal, depressive, and fibromyalgia symptoms. There was a mild improvement in the fibromyalgia impact score in those with mild-to-moderate vitamin D deficiency (10–25 ng/mL) compared to the placebo group. In patients with severe vitamin D deficiency (25OHD less than 10 ng/mL). Participants in this group who were treated in an unblinded fashion showed no symptom improvement when evaluated at 8 weeks or after a 1-year follow-up. A recent study from South Korea found a reduced risk of low muscle mass when serum 25-OHD levels improved from the insufficient to the acceptable range [19,20].

Our study aimed to examine whether the symptoms or patient complaints reflect changes in PTH and ALP, which signal biochemical and probably morphologic changes in the bones at certain vitamin D levels. Since we have noticed deficiency symptoms start only at a lower level (i.e., 25OHD below or around 15 ng/mL), we further reclassified vitamin D status in this study to the following: severe deficiency (25OHD < 10 ng/mL), moderate deficiency (10 to <15 ng/mL), mild deficiency (15 to <20 ng/mL), insufficiency (20 to <30 ng/mL), normal (30 to 100 ng/mL), and high (>100 ng/mL). We observed changes in PTH and ALP at different vitamin D levels to determine when changes in both parathyroid glands and bone occurred, as measured by PTH and ALP.

## 2. Materials and Methods

### 2.1. Participants

In this cohort, we collected 1617 samples over 5 years (2015–2019) and measured fasting 25OHD levels, intact PTH, and ALP. We excluded samples if laboratory results did not have all three variable results at the same time. We excluded patients with primary hyperparathyroidism, hypoparathyroidism, chronic kidney disease, and chronic liver disease, (only 1311 samples were analyzed). The majority of our patients are between 20 and 50 years of age. Since ALP values can be higher in younger populations, which might affect the results, we have excluded patients below 20 years old. Our patients are predominantly of Arab ethnicity (Saudi Arabia, Egypt, Sudan, Yemen, Jordan, Tunisia, Algeria, and Syria); women made up 56% of the respective samples, while men made up 44%.

### 2.2. Measurements

In this cohort, serum 25OHD was measured using an Elecsys^®^ Assay (Roche Diagnostics, Boston, MA, USA). In this assay, vitamin D3 (25OHD3) and vitamin D2 (25OHD2) bind to vitamin-D-binding protein as a capture protein.

Elecsys assay to determine PTH employs a sandwich test principle, where the N-terminal fragment (1–37) of the PTH reacts with a biotinylated monoclonal antibody and a ruthenium complex labeled monoclonal antibody reacts with the C-terminal fragment of the PTH hormone (38–84). For ALP determination, COBAS colorimetric assay was used.

Vitamin D was divided into six categories: severe deficiency (25OHD < 10 ng/mL), moderate deficiency (10 to <15 ng/mL), mild deficiency (15 to <20 ng/mL), insufficiency (20 to <30 ng/mL), normal (30 to 100 ng/mL), and high (>100 ng/mL). PTH was divided into three groups: low (<15 pg/mL), normal (15 to 65 pg/mL), and high (>65 pg/mL). Similarly, ALP was divided into three groups: low (<35 IU/L), normal (35 to 104 IU/L), and high (>104 IU/L).

### 2.3. Statistical Analysis

Statistical analyses were carried out using the software IBM SPSS Statistics, version 23. Descriptive analyses and frequencies were run for all scale variables for calculation of the mean, median, mode, standard deviation, and confidence intervals. Percentages were obtained for different categories. We compared the means of all three variables between males and females. We further compared ALP in females between 20 and 50 years old with that in those over 50. Chi-square test was applied to determine associations between categorical data for vitamin D, PTH, and ALK intervals. A *p* value of <0.05 was considered significant. Associations between serum 25OHD and PTH and ALP were assessed using Pearson’s correlation coefficient (two-tailed). SPSS was used to generate the coefficient rho. Normal distribution of data was checked, and due to nonparametric data, the Spearman rank test was applied, with a *p* value of <0.01 being considered significant.

## 3. Results

**Figure 1:**
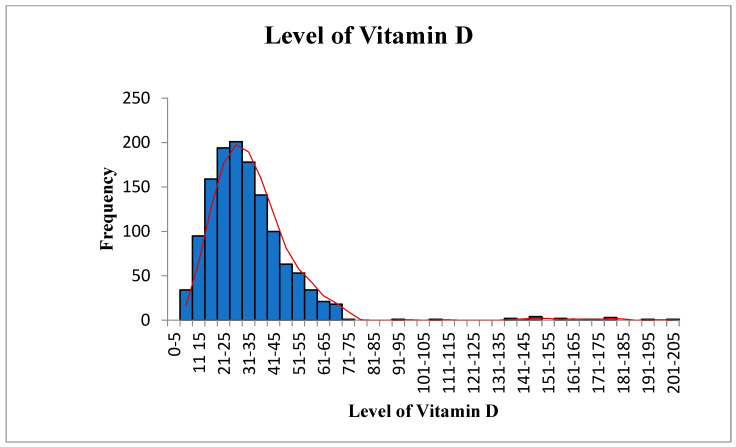
Vitamin D level.

**Figure 2:**
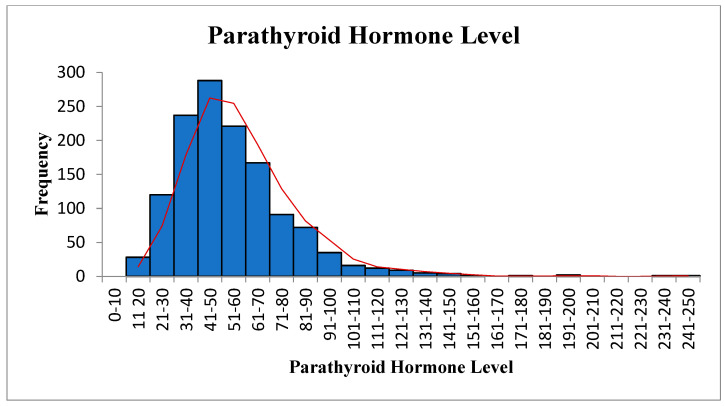
Parathyroid hormone level.

**Figure 3:**
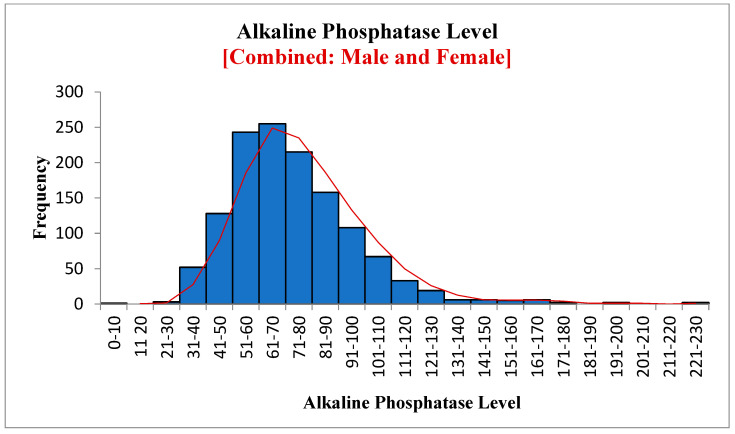
Alkaline phosphatase level.

Overall, 1311 samples were analyzed to assess the association between 25OHD, PTH, and ALP. Forty-four percent were male, and fifty-six percent were female. Table 1 shows the results of the descriptive analyses of all three variables. The mean (SD) value of 25OHD was 32.52 ± 20.14 (SE, 0.56; 95% confidence interval (CI), 31.43 to 33.61). The means were 32.86 in males and 32.25 in females, with no significant difference between them. The mean (SD) for PTH was 53.84 ± 24.13 (SE, 0.67; 95% CI, 52.53 to 55.15). The mean PTH value in males was 55.61 and 52.47 in females, with no statistically significant difference. The mean value of ALP was 73.14 ± 24.22 (SE, 0.67; 95% CI, 71.83 to 74.45), 74.09 in males and 72.51 in females, with no significant difference between them. The mean value of ALP was 72.53 in females between 20 and 50 years old and 72.45 in those over 50, with no statistically significant difference. Our study showed that only 3% of subjects had vitamin D below 10 ng/mL, 10% had below 15 ng/mL, 22% had below 20 ng/mL, and 42% had below 30 ng/mL. This is in comparison to a very recent large study (180,289 subjects), in which the overall prevalence of vitamin D deficiency status based on 25(OH)D level was as follows: 0.4% for <5 ng/mL, 12.5% for <10 ng/mL, 20.6% for <12 ng/mL, 49.4% for <20 ng/mL, and <75.3% for <30 ng/mL [21]. Furthermore, Figure 1, Figure 2 and Figure 3 show the data in graphical presentations that suggest that the levels of vitamin D, PTH, and ALK are skewed to the left. 

**Figure 4:**
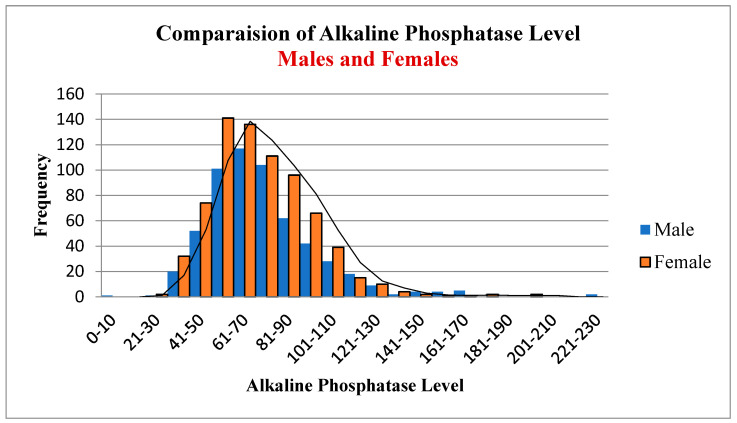
Alkaline phosphates in males and females.

**Figure 5:**
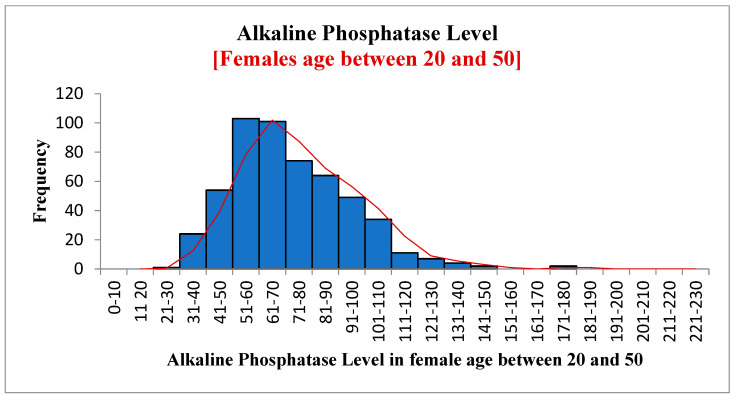
Alkaline phosphates in females aged 20–50.

**Figure 6:**
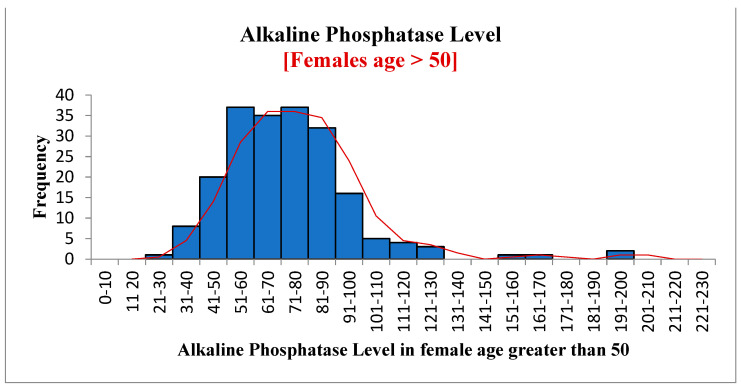
Alkaline phosphates in females over 50.

Figure 4, Figure 5 and Figure 6 show the frequency distribution of ALP in males and females, females between 20 and 50 years old, and females over 50. The mean ALP was 73.14 for all patients, 74.09 in males, 72.51 in females, 72.53 in females between 20 and 50 years old, and 72.45 in females over 50. There was no significant difference between these different groups. Statistics are available using the link given in the data availability statement. 

Table 2 shows the numbers and percentages of the different vitamin D levels, PTH, and ALK in categories from deficient to normal. The results show that many patients (i.e., 47%) had a normal level of 25OHD, followed by 30% who had insufficiency, 12% who had a mild deficiency, 7% who had a moderate deficiency, and 3% who had severe deficiency. Levels of PTH were also found to be normal in 74% of samples, followed by 25% with increased levels and 1% with low PTH levels. ALP levels were normal in 91% of the samples, whereas 9% were high, and 1% exhibited a low level of ALP.

As shown in Table 3, 25OHD was associated with PTH and ALK. 25OHD was significantly associated (*p* < 0.001) with PTH as well as ALP (*p* < 0.001). The table further reveals that the participants with severe deficiency of vitamin D (*n* = 34) had increased levels of PTH (65%) and ALK (21%). In contrast, the participants with moderate 25OHD deficiency (*n* = 95) had 41% normal and 58% increased PTH levels. However, in the same category, ALP was normal in most cases (84%). In mild 25OHD deficiency (*n* = 159), most patients (70%) had normal PTH, and 87% had normal levels of ALK. Similarly, in those with insufficient vitamin D levels (*n* = 395), PTH was normal in (73.6%), and ALP was normal in 92% of the participants.

The data shown in Table 4 suggest that individuals with severe vitamin D deficiency have high mean PTH (85.56 ± 38.80 pg/mL) and mean ALP (82.61 ± 30.12 IU/L) levels. In contrast, those with vitamin D levels above 100 ng/mL have lower mean PTH (38.49 ± 20.34 pg/mL) and mean ALP (55.52 ± 20.15 IU/L) levels.

The correlation is summarized in Table 5, which shows a negative correlation between 25OHD and PTH (*Rs* = −0.24). The value further suggests a correlation that weakly influences two variables (i.e., as the level of vitamin D decreases, the level of PTH will increase). However, although this correlation is weak, it is highly significant (*p* < 0.001). Moreover, there was a negative weak (*Rs* = −0.18) but significant (*p* < 0.001) correlation between 25OHD and ALP. As expected, there was a positive, weak (*Rs* = −0.11), but significant (*p* < 0.001) correlation between ALP and PTH.

**Figure 7:**
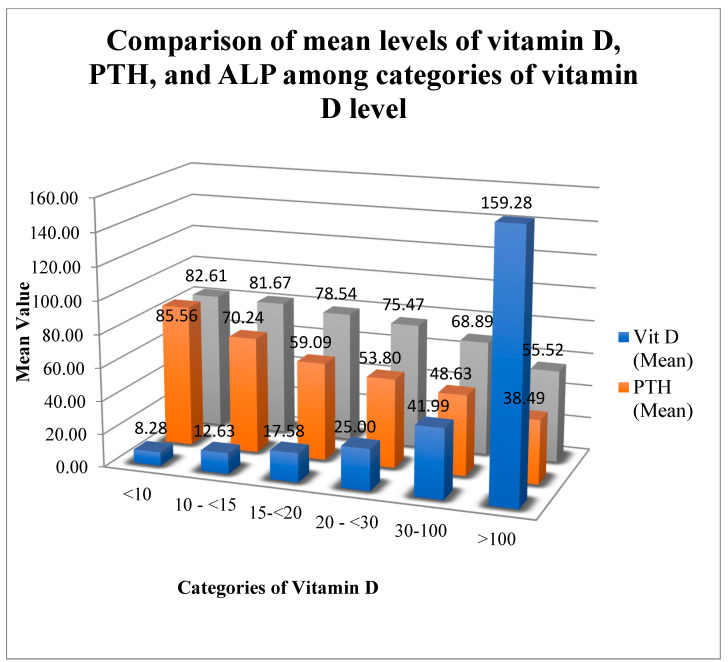
Comparison of mean levels of vitamin D, PTH, and ALP between categories of vitamin D level.

Figure 7 shows the results of a comparison of the mean values of vitamin D, PTH, and ALK according to the different categories of vitamin D. The figure further reveals that as the mean level of vitamin D decreases, both PTH and ALK mean levels significantly increase; however, as the mean level of vitamin D increases, the levels of PTH and ALK decrease considerably. 

In severe vitamin D deficiency (<10 ng/mL), PTH was higher than normal in 65% of patients, and ALP was high in 21% of the patients. In patients with moderate vitamin D deficiency (10 to <15 ng/mL), PTH was high in 58% and ALP was high in 16%. In patients with mild vitamin D deficiency (15 to <20 ng/mL), PTH was high in 30%, and ALP was high in 13%. In the vitamin D insufficiency group (20 to <30 ng/mL), PTH was high in 25.5% and ALP in 8%. In patients with normal vitamin D levels (30 to 100 ng/mL), 83% had normal PTH, 1% had low PTH and 16% had high PTH. In the same group in which vitamin D was above 30 ng/mL, ALP was normal in 93% of the patients, high in 6%, and low in 1% of the patients.

## 4. Discussions

Vitamin D deficiency is common worldwide, including in our area in the Middle East. Inadequate exposure to sunlight is considered the major cause of vitamin D deficiency [22,23]. Although we have many sunny days all year long (around 320 sunny days per year), the prevalence of vitamin D deficiency is very high. Certain factors are responsible for this, including but not limited to traditional clothing influenced by environmental, religious, and social beliefs due to which women cover all of their bodies when going outside their houses (including their faces, hair, and sometimes hands), while men cover most of their bodies except their faces and hands to avoid the direct effect of hot sun rays. Other factors include shift work, indoor work, a lack of dairy products, and the application of sunscreen [24]. Most recently, the urban lifestyle has changed some of our social habits, including sleeping during the day and staying up all night as in summer holidays; however, this practice is a routine lifestyle for most unemployed homemakers, especially those who live in apartments. Other factors include an increase in the number of hours spent indoors compared to outdoors due to smart devices, televisions, and a lack of outdoor recreational spaces; downsizing of the living space has also occurred, since a large percentage of families live in apartments in big cities with no outdoor spaces compared to larger houses with front or back yards in smaller cities, which allow more sun exposure. People with darker skin possess natural sun protection. However, compared to the white population, they need at least 3–5 times more sun exposure to produce the same amount of vitamin D [25,26]. We believe our Arab population with brown skin absorbs sunlight in an amount between these two races. Seasonal variations in vitamin D have been reported [27]. We may not have large seasonal variation in our area because we have sunny days all year round, but considering this in greater detail in the future is worthwhile.

Endocrine Society guidelines suggest treating patients with vitamin D insufficiency, defined as a 25OHD of 21 to 30 ng/mL, or deficiency, defined as a 25OHD <20 ng/mL. Their rationale was increased efficiency of calcium absorption when 25OHD levels increase above 20 ng/mL [28] and plateauing of PTH when vitamin D levels are above 30 ng/mL, among other clinical benefits [3,5,6].

In a recent study, there were biochemical changes (low Ca++, low PO4, high PTH, and high ALP) when 25OHD was below 10 ng/mL, however, there was no biochemical evidence when the levels were between 20–30 mg/mL to justify labeling patients with vitamin D insufficiency or to justify vitamin D supplementation in this category [16].

Based on our clinical observations, most patients complain of symptoms only when their levels are around 15 ng/mL or lower, where the lower the levels, the greater the severity. Many patients refuse treatment when they do not have symptoms, and some will accept taking the prescription but not follow our recommendations because they are symptom-free. These observations made us question whether the term “vitamin D insufficiency” is valid. We looked at changes in PTH and ALP at different vitamin D levels to see if there are any biochemical changes when 25OHD is in the insufficient range. According to the Endocrine Society guidelines definition, vitamin D deficiency is defined as a 25OHD of <20 ng/mL, but since most patients with vitamin D > 15 ng/mL are asymptomatic as clinically observed, we further reclassified patients in this group into three groups: mild deficiency, 25OHD between 15–<20 ng/mL; moderate deficiency, 25OHD between 10 and <15 ng/mL; and severe deficiency, 25OHD < 10 ng/mL. As expected, and as shown in previous studies [4,29,30,31,32,33], our study showed an inverse and significant relationship between vitamin D on the one hand and PTH and ALP on the other. The lower the vitamin D level, the higher both PTH and ALP levels. In those with mild, moderate, or severe vitamin D deficiency, PTH was higher than normal in 30%, 58%, and 65%, respectively. ALP was higher than normal in 13%, 16%, and 21%, respectively. Another study found that only 34% of participants had secondary hyperparathyroidism, and only 6% had elevated ALP when 25OHD was below 10 ng/mL [16]. In the vitamin D insufficiency group (20 to <30 ng/mL), PTH was high in 25.5%, and ALP was high in 8%. In patients with normal vitamin D levels, 83% had normal PTH, 1% had low PTH, and 16% had high PTH. This elevation in PTH while having normal vitamin D is unexplained but could indicate a lag time between correction of vitamin D after treatment and normalization of PTH since patients with primary hyperparathyroidism and CKD have been excluded. In this same group in which vitamin D was above 30 ng/mL, ALP was normal in 93%, high in 6%, and low in 1% of patients The elevation of ALP might suggest continuous bone remodeling, or it may take time for ALP to normalize after normalization of vitamin D if the patient was previously deficient before treatment.

## 5. Limitations of the Study

Our current study has some limitations, including a, lack of symptom details, data on seasonal variation in vitamin D, and medication intake status. In addition, we have a limited number of patients with severe and moderate vitamin D deficiency.

## 6. Conclusions and Future Work

As expected, there was a significant inverse correlation between 25OHD, PTH, and ALP. There was no significant difference between the means of 25OHD, PTH, and ALP between males and females. Additionally, there was no significant difference in ALP mean between females between 20 and 50 years old and those over 50. PTH and ALP were normal in most of the patients with mild vitamin D deficiency and those with vitamin D insufficiency, which possibly explains the lack of symptoms in those patients whose vitamin D levels were above 15 ng/mL. Searching for effects on bones by means other than ALP and PTH may help in the search for silent bone changes to validate vitamin D supplementation in these populations. Correction for these limitations would enable more confident conclusions to be drawn in future research. Our future research will include demographic data, calcium and phosphorous, bone remodeling markers, presence/absence of symptoms with symptoms details, vitamin D supplementation status, and seasonal variation. 

## Figures and Tables

**Table 1:** Descriptive analysis (N = 1311).

Variables			Statistic	Std. Error
25OHD (ng/mL)	Mean		32.52	0.56
	95% Confidence Interval for Mean	Lower Bound	31.43	
		Upper Bound	33.61	
	5% Trimmed Mean		30.88	
	Median		29.30	
	Variance		405.75	
	Std. Deviation		20.14	
	Range		197	
	Interquartile Range		17.77	
PTH (pg/mL)	Mean		53.84	0.67
	95% Confidence Interval for Mean	Lower Bound	52.53	
		Upper Bound	55.15	
	5% Trimmed Mean		52.54	
	Median		49.20	
	Variance		582.83	
	Std. Deviation		24.13	
	Range		238	
	Interquartile Range		17.77	
ALK (U/L)	Mean		73.14	0.67
	95% Confidence Interval for Mean	Lower Bound	71.83	
		Upper Bound	74.45	
	5% Trimmed Mean		71.79	
	Median		69.0	
	Variance		586.83	
	Std. Deviation		24.22	
	Range		221	
	Interquartile Range		30.65	

**Table 2:** Frequency and percentage of vitamin D, ALK, and PTH levels (N = 1311).

Classification	Levels	Frequency (%)
**25OHD (ng/mL)**		
Severe deficiency	<10	34 (3)
Moderate deficiency	10–<15	95 (7)
Mild deficiency	15–<20	159 (12)
Insufficient	20–<30	395 (30)
Normal	31–100	610 (47)
Increased	>101	18 (1)
**PTH (pg/mL)**		
Deficient	<15	8 (1)
Normal	15–65	976 (74)
Increased	>65	327 (25)
**ALP (IU/L)**		
Low	<35	9 (1)
Normal	35–104	1188 (91)
Increased	>104	114 (9)

**Table 3:** Association between vitamin D and PTH and ALK (*n* = 1311).

**25OHD (ng/mL)**	**PTH (pg/mL)**				***p* Value**
	**<15 pg/mL** (Deficient) # Of patients (%)	**15–65 pg/mL** (Normal) # Of patients (%)	**>65 pg/mL** (Increased) # Of patients (%)	**Total (%)**	
Severe deficiency (<10)	0	12 (35)	22 (65)	34 (100)	<0.001
Moderate deficiency (10–<15)	1 (1)	39 (41)	55 (58)	95 (100)	
Mild deficiency (15–<20)	0	111 (70)	48 (30)	159 (100)	
Insufficient (20–<30)	3 (0.7)	291 (73.6)	101 (25.5)	395 (100)	
Normal (30–100)	4 (1)	506 (83)	100 (16)	610 (100)	
Increased (>100)	0	17 (94)	1 (6)	18 (100)	
**25OHD (ng/mL)**	**ALP (IU/L)**				***p* Value**
	**<35 IU/L** (Low) # Of patients (%)	**35–104 IU/L** (Normal) # Of patients (%)	**>104 IU/L** (Increased) # Of patients (%)	**Total (%)**	
Severe deficiency (<10)	0	27 (79)	7 (21)	34 (100)	<0.001
Moderate deficiency (10–<15)	0	80 (84)	15 (16)	95 (100)	
Mild deficiency (15–<20)	0	139 (87)	20 (13)	159 (100)	
Insufficient (20–<30)	0	362 (92)	33 (8)	395 (100)	
Normal (30–100)	8 (1)	563(93)	39 (6)	610 (100)	
Increased (>100)	1 (6)	17 (94)	0	18 (100)	

**Table 4:** Mean and SD of PTH and ALP according to vitamin D interval (classification).

Vitamin D (ng/mL)	PTH (Mean ± SD)	ALP (Mean ± SD)
Severe deficiency (<10)	85.56 ± 38.80	82.61 ± 30.12
Moderate deficiency (10–<15)	70.24 ± 29.90	81.67 ± 28.06
Mild deficiency (15–< 20)	59.09 ± 28.24	78.54 ± 24.36
Insufficient (20–<30)	53.80 ± 21.20	75.47 ± 24.26
Normal (30–100)	48.63 ± 19.63	68.89 ± 22.15
Increased (>100)	38.49 ± 20.34	55.52 ± 20.15
**Total**	**53.84 ± 24.14**	**73.14 ± 24.22**

**Table 5:** Correlation between vitamin D and PTH and ALK (n = 1311).

Variables		Level of Vitamin D (ng/mL)	Level of PTH (pg/mL)	Level of ALK (IU/L)
**Level of vitamin D (ng/mL)**	Correlation Coefficient (*Rs*)	1.000	−0.24 *	−0.18 *
	Sig. (2-tailed)	----	<0.001	<0.001
**Level of PTH (pg/mL)**	Correlation Coefficient (*Rs*)	−0.24 **	1.000	0.11 **
	Sig. (2-tailed)	<0.001	.	<0.001
**Level of ALP** **(IU/L)**	Correlation Coefficient (*Rs*)	−0.18 **	0.11 **	1.000
	Sig. (2-tailed)	<0.001	<0.001	----

* Correlation is significant at the 0.01 level (2-tailed). ** Correlation is significant at the 0.01 level (2-tailed).

## Data Availability

All the data are available within the manuscript, and the data files can be downloaded using the link https://www.dropbox.com/scl/fo/jqganeyixd63ujtfkczwb/h?dl=0&rlkey=p6jdzmqp0lpb5u1vi5v0a98ba, accessed on 1 November 2022.
